# Granulocyte-macrophage colony-stimulating factor may contribute to spondyloarthritis development in HLA-B27 transgenic rat by affecting conventional dendritic cells function

**DOI:** 10.1186/s13075-025-03586-9

**Published:** 2025-06-13

**Authors:** Amel Ait Ali Said, Chiara Rizzo, Hendrick Mambu Mambueni, Félicie Costantino, Maxime Breban, Simon Glatigny

**Affiliations:** 1 Université Paris Saclay, Université de Versailles St Quentin en Yvelines, Inserm, Infection et Inflammation, Montigny le Btx INSERM UMR1173, UFR Simone Veil, Versailles-Saint-Quentin University, Inserm, France; 2 INFLAMEX, Laboratoire d’Excellence, Université Paris Cité, Paris, France; 3 Rheumatology Division, Ambroise Paré Hospital (AP-HP), Boulogne- Billancourt, France

## Abstract

**Background:**

Spondyloarthritis (SpA) is a chronic inflammatory disorder with axial and peripheral manifestations. A strong association between HLA-B27 and SpA has been known for more than 50 years. Remarkably, HLA-B27 and human β2-microglubulin transgenic rats (B27 rat) develop manifestations recapitulating SpA, referred to as rat SpA. Antigen-presenting cells such as dendritic cells (DC) and CD4 T cells are mandatory to develop rat SpA. Serum levels of granulocyte macrophage-colony stimulating factor (GM-CSF), a key growth factor for DC generation and functions, are significantly increased during SpA. Conventional (c)DCs can be divided in two subsets implicated either in immune tolerance (cDC1) or in adaptive immune responses induction (cDC2). In this study, we aimed to determine the influence of GM-CSF on cDC subsets functions linked to T cell activation and differentiation, in the B27 rat model.

**Methods:**

cDC subsets were isolated from spleens of B27 and nontransgenic (NTG) rats, primed with GM-CSF and tested for their ability to support CD4h T cell differentiation. RNA sequencing was performed on GM-CSF-primed cDC subsets.

**Results:**

GM-CSF-primed cDC2 from B27 rat were strong inducers of TNF-producing proinflammatory CD4^+^ T cells. In contrast, whereas control cDC1 required GM-CSF to support T cell proliferation, HLA-B27^+^ cDC1 primed with GM-CSF failed to do so. RNA sequencing analysis demonstrated that HLA-B27 expression promoted endoplasmic reticulum stress and unfolded protein response in both cDC subsets. In addition, HLA-B27 expression promoted inflammatory cytokine synthesis by cDC2 and a signature interfering with regulation of cell adhesion and activation in cDC1.

**Conclusion:**

Altogether, our study reveals a dual role of GM-CSF during SpA. In one hand, GM-CSF promotes proinflammatory functions of cDC2. On the other hand, GM-CSF is required for cDC1 to induce T cell proliferation, and those functions are blunted by HLA-B27 expression.

**Supplementary Information:**

The online version contains supplementary material available at 10.1186/s13075-025-03586-9.

## Introduction

Spondyloarthritis (SpA) is a chronic inflammatory rheumatism characterized by axial and peripheral arthritis frequently combined with extra-articular features. Its estimated prevalence is between 0.2 and 1.6% worldwide [1]. SpA development is believed to depend on both genetic and environmental factors. The class I-major histocompatibility complex (MHC) human leucocyte antigen B27 (HLA-B27) allele is the strongest genetic factor of susceptibility [2]. Indeed, 60 to 80% of SpA patients are HLA-B27^+ 2^. Despite more than 50 years of study, the mechanisms linking HLA-B27 to SpA development are not yet fully understood. The proof of a pathogenic function of HLA-B27 during SpA was provided by the production of HLA-B27 transgenic rats [3]. Several lines of rats transgenic for HLA-B27 and human β2-microglobulin (hβ2m) (B27 rats) develop spontaneously a disease mimicking human SpA referred to as “rat SpA” [3]. Cell transfer experiments showed that HLA-B27^+^ bone marrow (BM)-derived cells and CD4^+^ T cells were both required for rat SpA development [4]. A prominent expansion of proinflammatory T helper 17 (Th17) cells producing IL-17 and TNF was observed in B27 rats in parallel with disease development [5]. Moreover, it has been established that germ-free B27 rats do not develop SpA, highlighting the crucial role of microbiota in SpA pathogenesis [6].

DCs are BM-derived professional antigen-presenting cells (APCs) sensing changes in their environment that are critical to trigger CD4^+^ T cell proliferation and differentiation. DCs can be divided into two main categories: plasmacytoid dendritic cells producing high amount of type I IFN upon viral infection and conventional dendritic cells (cDC) specialized in antigen presentation to T cells. In rat, two subsets of cDCs have been identified based on CD103 and CD4 expression: cDC1 (CD103^+^ CD4^−^) involved predominantly in immune tolerance and cross-presentation to CD8^+^ T cells and cDC2 (CD103^+^ CD4^+^) which are the most efficient APCs for CD4^+^ T cell activation and polarization [7]. We previously described altered functions of cDCs during both SpA and rat SpA characterized by a reduced ability to support T cell proliferation [8–10]. Recent studies identifying specific roles of each cDC subset suggested that the alterations mediated by HLA-B27 expression could be subset-specific. For instance, the frequency and absolute numbers of XCR1-expressing cDC1 was decreased during rat SpA in B27 rats [11] whereas cDC2 displayed altered cytoskeleton dynamics and reverse interferon (IFN) signature [12]. Thus, a closer examination of cDC subsets function during SpA is required to understand how they may contribute to the striking proinflammatory CD4^+^ T cells expansion [5].

Granulocyte-macrophage colony-stimulating factor (GM-CSF) is a growth factor important for myeloid cells function that is produced by a variety of cells, including proinflammatory T cells [13]. GM-CSF is a pleiotropic cytokine stimulating differentiation, proliferation and survival of bone marrow progenitor cells and innate immune cells including cDCs [14]. These effects enhance innate and adaptive immune responses, increase tissue inflammation, and are linked to inflammatory diseases pathogenesis [15]. During SpA, GM-CSF serum levels and production by peripheral blood mononuclear cells (PBMCs) are significantly increased [16, 17]. This observation led to the hypothesis that GM-CSF could be pathogenic during SpA [16]. Accordingly, clinical trial targeting GM-CSF was performed in SpA but did not result in significant improvement, suggesting a more complex role of GM-CSF during SpA [18]. Thus, the aim of our study was to address the role of GM-CSF on XCR1^+^ cDC1 and cDC2 functions during rat SpA.

## Materials and methods

### Animals

The SpA-prone B27 rats of the 33 − 3 line bearing 55 copies of HLA-B*27:05 and 28 copies of hβ2m and the healthy B7 rats of the 120-4 line bearing 52 copies of HLA-B*07:02 and 26 copies of hβ_2_m on a Fisher (F344) background were bred and maintained under conventional conditions. We used B27 rats aged of 3 weeks (premorbid) or 6 months (with established SpA). Age-matched nontransgenic (NTG) littermates and B7 rats were used as controls. The 33 − 3 line was backcrossed onto F344 rats homozygous for the *rnu* allele (*Foxn1*-deficient, Charles River) which confers thymic aplasia, to generate nude (*rnu/rnu*) B27 rats protected from SpA development. Study procedures were approved by ethical committee and by the French Ministry of Agriculture (APAFIS#28596-2020111317039573 v6).

### Isolation of splenic cDC subsets

Splenic cDCs were isolated as previously described [11]. Briefly, spleens were collected, perfused with collagenase D (2 mg/ml), minced into pieces and digested for 20 min at 37 °C. EDTA 0.5 mM was added to block the reaction. Cell suspensions were prepared, and low-density cells were isolated after centrifugation on a 14.5% Nycodenz gradient (Nycomed). Cells at the interface were carefully collected and cDCs were purified using anti-CD103-coated microbeads (clone OX62, Miltenyi Biotec) and an autoMACS^®^ Pro Separator (Miltenyi Biotec). cDC2 and XCR1^+^ cDC1 were isolated on Aria III cell sorter after staining with a cocktail of fluorochrome-conjugated monoclonal antibodies (mAbs) for 20 min at 4 °C in dark. The following mAbs were used: fluorescein isothiocyanate (FITC)-conjugated anti-rat TCRαβ (R73) and anti-rat CD45RA (OX33) to remove T and B cell contaminants, Alexa Fluor 647 (AF647)-conjugated anti-rat CD103, phycoerythrin (PE)-conjugated anti-rat XCR1 and peridinin-chlorophyll-protein eFluor 710 (PerCp-e710)-conjugated anti-rat CD4. The sorting gating strategy used is represented in Supplementary Fig. 1. Purity of cDC subsets was ≥ 99% and all sorted cells were alive (data not shown).

### cDC priming with GM-CSF

Purified splenic cDC2 and XCR1^+^ cDC1 were cultured overnight in 96-wells round bottom plate (10^4^ cells per well) in complete RPMI 1640 medium GlutaMax I (Life Technologies) containing 10% fetal calf serum, streptomycin (100 µg/ml), 2% sodium pyruvate, 0.05 mM 2-mercaptoethanol, and 5 mM HEPES, in the presence or not of recombinant rat GM-CSF (100 ng/mL, Biolegend). GM-CSF signaling pathways were evaluated by adding specific inhibitors at saturating concentrations during the overnight culture. The inhibitors targeted extracellular signal-regulated kinases (ERKi: UO126; 10 µM), p-38 kinase (SB203580: 10 µM), c-Jun N-terminal kinases (JNKi: SP600125; 20 µM), Janus kinases 1/3 (JAK1/3i: Tofacitinib; 60 nM) and JAK2 (JAK2i: AG-490;10 µg/mL).

### cDC-T cell coculture

Purified splenic cDC2 and XCR1^+^ cDC1 (10^4^ cells per well) were primed overnight with GM-CSF as described above. The next day, CD4^+^CD25^−^CD62L^High^ naïve T cells were isolated from mesenteric lymph nodes (mLN) of NTG rats, using appropriate gating strategy with an Aria III cell sorter (BD Biosciences). Naïve T cells were labelled with cell trace violet (CTV; 5 μm, ThermoFisher) according to the manufacturer’s instructions and cultured for 6 days with GM-CSF-primed cDCs at a ratio of 1 DC for 5 T cells (5 × 10^4^ T cells per well) in the presence of anti-TCRab mAb (R73: 1 µg/mL).

### Flow cytometry

After cDCs isolation, cells were washed and stained with the following Abs: FITC-conjugated anti-rat TCRαβ, FITC-conjugated anti-rat CD45RA, AF647-conjugated anti-rat CD103, PE-cyanine-7 (Pe-Cy7)-conjugated anti-rat CD4, brilliant violet 510 (BV510)-conjugated anti-rat XCR1, BV650-conjugated anti-rat RT1B (rat class II MHC), PE-conjugated anti-rat CD80. After 30 min labelling at 4 °C in the dark, cells were washed and resuspended in 300 µl of phosphate buffered saline (PBS) 1X before acquisition using a LSRII FORTESSA flow cytometer (Becton Dickinson).

After XCR1^+^ cDC1 and cDC2 maturation with GM-CSF, cells were washed and labeled with Live/dead APC e780 (ThermoFisher) to exclude dead cells. Cells were then washed and stained with saturating mAb cocktail for 30 min at 4 °C in the dark. The following mAbs were used: BV650-conjugated anti-rat RT1B, BV605-conjugated anti-rat CD86 and PE-conjugated anti-rat CD80.

At day 6 of DC-T cell co-culture, cells were stimulated with phorbol myristate acetate and ionomycin (both at 500 ng/mL) in the presence of brefeldin A (10 µg/mL) for 4 h. Cells were stained with LIVE-DEAD™ fixable reagent (ThermoFisher) to exclude dead cells prior to fixation and permeabilization with Foxp3 Transcription Factor Staining Buffer Kit according to the manufacturer instructions (Tonbo Biosciences). Cells were stained with PerCp-e710-conjugaetd anti-CD4 and PeCy7-conjugated anti-TNF Abs. After staining, cells were washed, resuspended before acquisition on a LSR II Fortessa flow cytometer. Data were analyzed using FlowJo software version 10.6.2 (TreeStar). Proliferation of T cells was assessed by evaluating the dilution of CTV in live CD4^+^ T cells.

### Sequencing of cDCs RNA

Purified splenic cDC2 and XCR1^+^ cDC1 from 6 sex-matched euthymic adult (> 6 months-old) NTG and B27 rats were cultured overnight in 96-wells round bottom plate (at a concentration of 10^5^ cells per mL) in complete media containing recombinant rat GM-CSF (100 ng/mL). After priming overnight with GM-CSF, XCR1^+^ cDC1 and cDC2 were washed 5 min at 1,200 rpm to remove dead cells and total RNA was purified using Monarch Total RNA Miniprep Kit (New England Biolabs). Samples were qualified on LabChip GX (Caliper-Perkin Elmer) and those with RNA integrity number > 7 were selected for sequencing. Libraries were prepared using NEBNext rRNA Depletion Kit and NEBNext Ultra II Directional RN Library Prep Kit and sequenced at the genomic platform o-f Faculty Simone Veil of Versailles-Saint-Quentin University on an Illumina NextSeq 500/550, yielding paired-end 75 bases sequences. FASTQ files were trimmed using fastp version 0.19.5 [19] and then aligned to the rat genome Rnor_6.0.101 using STAR version 2.7.1 [20]. Finally reads were counted using featureCounts [21].

### Statistical analysis

Data are presented as the mean ± SEM or box and whiskers with median and the box covering the interquartile interval. Comparisons were performed by two-way ANOVA followed by correction for multiple comparisons using Tukey’s test in GraphPad Prism version 9. RNA sequencing differential analysis between NTG and B27 rats was performed in R (R Foundation for Statistical Computing) using DESeq2 version 1.42.0 with sex and batch effect included in the design [22]. Threshold of adjusted p-value < 0.05 and log2 fold change > 1 were used to determine significantly differentially expressed genes (DEG). Volcano plots were generated with Enhanced Volcano. Functional enrichment analysis of DEGs was performed, using biological processes identified using clusterProfiler version 4.10.0 [23] and visualized with enrichPlot. Enrichment scores were calculated as the ratios of observed significant genes to the expected number of significant genes, where the expected count is derived by multiplying the background gene ratio by the total number of significant genes analyzed. The Venn diagram showing each cDC specificity of DEGs was generated using ggvenn.

## Results

### B27 rat cDCs primed with GM-CSF induce altered CD4^+^ T cell response

**Fig. 1:**
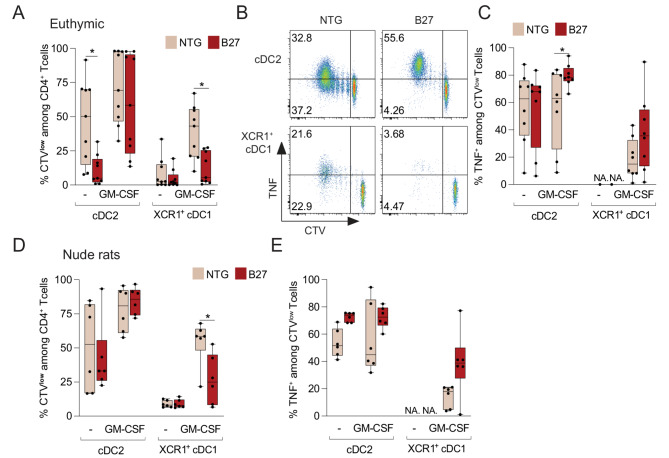
GM-CSF-primed HLA-B27^+^ cDCs lead to aberrant proinflammatory CD4^+^ T cell responses. cDC2 and XCR1^+^ cDC1 were isolated from euthymic (*n* = 11 rats per group) or nude (*n* = 6 rats per group) NTG and B27 rats and cultured overnight with or without GM-CSF. Then, primed cDCs were tested for their ability to support NTG naïve CD4^+^ T cells proliferation and differentiation in the presence of anti-TCRab mAb. (**A** and **D**) The box and whiskers graphs show the frequency of proliferating T cells after culture with untreated (-) or GM-CSF-primed cDCs from euthymic (**A**) or nude (**D**) NTG (beige) and B27 rats (red). (**B**) The representative plots show T cell proliferation evaluated by CTV dilution and TNF production gated on live T cells cultured with GM-CSF primed cDC2 (top) or XCR1^+^ cDC1 (bottom) isolated from NTG (left) or euthymic B27 rat with established SpA (right). (**C** and **E**) The graphs show the frequency of TNF^+^ cells among proliferating T cells after co-culture with GM-CSF-primed cDCs from euthymic (**C**) or nude (**E**) NTG and B27 rats. *n* > 5 rats per group *p < 0.05

As GM-CSF is an important factor for cDCs, we first tested if it was required for cDC subsets to trigger T cell proliferation. To this end, splenic cDC2 (CD103^+^CD4^+^) and XCR1^+^ cDC1 (XCR1^+^CD103^+^CD4^-^) isolated from NTG rats were exposed or not overnight to GM-CSF and used to stimulate CTV-labelled naïve CD4^+^ T cells from NTG rat. The strongest levels of T cells proliferation were reached when cDCs were primed with GM-CSF (Fig. 1A). Importantly, whereas cDC2 supported some T cell proliferation, XCR1^+^ cDC1 were unable to support T cell proliferation in the absence of GM-CSF (Fig. 1A). After determining that GM-CSF was required for optimal APC function of cDCs, we examined how GM-CSF-primed cDC subsets from B27 rats supported naïve CD4^+^ T cell proliferation and differentiation. GM-CSF-primed cDC2 from euthymic NTG and B27 rats induced equally strong T cell proliferation but cDC2 from B27 rats were more dependent on GM-CSF for this function (Figs. 1A and B). In contrast to cDC2, GM-CSF-primed XCR1^+^ cDC1 from NTG rats induced weaker T cell proliferation, whereas those isolated from B27 rats barely supported T cell proliferation (Figs. 1A and B). In addition, GM-CSF-primed XCR1^+^ cDC1 supported more regulatory T cell (Treg) differentiation than cDC2 corroborating their tolerogenic functions (Supplementary Fig. 2).

Intracellular cytokine staining showed that proliferating T cells produced significantly more TNF after co-culture with HLA-B27^+^ than control NTG cDC2 (Fig. 1B, C and Supplementary Fig. 3), indicating that HLA-B27^+^ cDC2 favor proinflammatory differentiation bias in CD4^+^ T cells. A similar trend was observed for the few T cells having proliferated in co-culture with HLA-B27^+^ XCR1^+^ cDC1 (Fig. 1C). Intracellular production of IL-17 by CD4^+^ T cells was not detected in those cultures. As altered functions of HLA-B27^+^ cDCs might be secondary to the inflammatory environment of rat SpA, we performed similar experiments with cDCs isolated from athymic nude B27 rats (protected from disease development) and obtained comparable results with the most remarkable decreased ability of GM-CSF-primed HLA-B27^+^ XCR1^+^ cDC1 to induce T cell proliferation (Figs. 1D and E). Altogether, these data show that the priming of HLA-B27^+^ cDCs with GM-CSF leads to proinflammatory CD4^+^ T cells differentiation bias (cDC2) and dysregulated function of XCR1^+^ cDC1. Moreover, those cDC dysfunctions are associated with HLA-B27 expression rather than secondary to rat SpA.

### Altered response of HLA-B27^+^ XCR1^+^ cDC1 to GM-CSF precedes rat SpA development

**Fig. 2:**
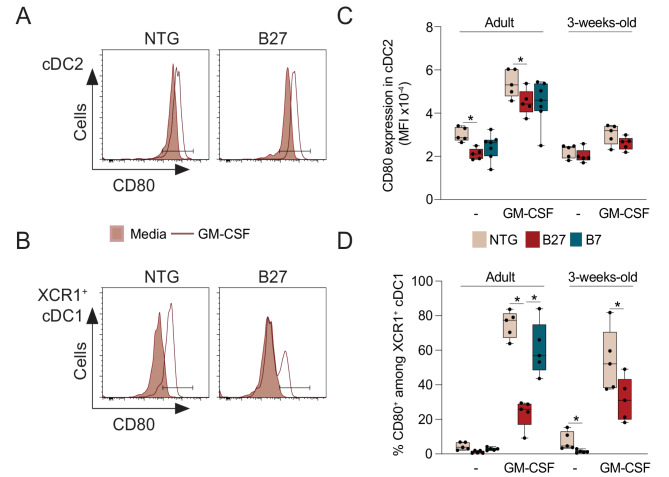
Altered response of cDCs to GM-CSF in B27-rats. cDC2 (**A**,**C**) and XCR1^+^ cDC1 (**B**,**D**) were isolated from adult NTG, B27 (with established SpA) and B7 adult rats or 3-weeks-old NTG and B27 rats and cultured overnight in absence (-) or presence of GM-CSF. cDCs maturation reflected by CD80 expression was evaluated at the end of the culture by flow cytometry. Representative plots showing the expression of CD80 on cDC2 (**A**) and XCR1^+^ cDC1 (**B**) from adult rats respectively. (**C**,**D**) The expression of CD80 was determined according to FMO staining (Supplementary Fig. 4). The graphs show the mean fluorescence intensity (MFI) of CD80 expression on cDC2 (**C**) and the frequency of CD80^+^ cells among XCR1^+^ cDC1 (**D**) from adult and 3-weeks-old NTG (beige), B27 rats (red) and/or B7 (magenta) rats. Results are shown as box and whiskers graph. *n* = 5 rats per group; *: *p* < 0.05

Having shown that GM-CSF primed HLA-B27^+^ cDCs led to dysregulated CD4^+^ T cell responses, we analyzed upstream events by evaluating cDC maturation after priming with GM-CSF. We focused on expression levels of surface molecules required for CD4^+^ T cell activation and differentiation, such as the costimulatory molecules CD80 and CD86, and the MHC class II molecule RT1B. CD80 and CD86 were expressed constitutively in cDC2 and were upregulated upon addition of GM-CSF both in control and B27 conditions (Figs. 2A, C and Supplementary Figs. 4 and 5). However, HLA-B27^+^ cDC2 expressed significantly lower levels of CD80 than controls NTG or HLA-B7^+^ (not associated to SpA) cDC2 either in the absence of exogenous stimulus or after priming with GM-CSF (Figs. 2A and C). In contrast, RT1B expression was not modified by exposure to GM-CSF but, similarly to CD80, was lower in HLA-B27^+^ cDC2 (Supplementary Fig. 5). In sharp contrast to cDC2, only a minority of XCR1^+^ cDC1 expressed CD80 without stimulation (Figs. 2B and D). Priming with GM-CSF led to substantial CD80 expression by controls XCR1^+^ cDC1 but such induction was significantly blunted in HLA-B27^+^ XCR1^+^ cDC1 (Figs. 2B and D). Similar reduced frequencies of CD86 and RT1B^High^ in HLA-B27^+^ XCR1^+^ cDC1 were also observed in the absence or presence of GM-CSF (Supplementary Fig. 5).

Then, we addressed if those dysfunctions were already present before disease development. As observed in adult rats, all cDC2 expressed CD80, however CD80 expression levels were lower in 3-weeks-old rats (Fig. 2C). At this young age, addition of GM-CSF did not increase CD80 nor CD86 expression levels in NTG rats (Fig. 2C and Supplementary Fig. 5). No changes in the expression levels of CD80 or CD86 were observed in HLA-B27^+^ cDC2 after GM-CSF treatment. Similarly to adult rat findings, CD80 was expressed by very few XCR1^+^ cDC1 and was strongly induced after GM-CSF treatment in 3-weeks-old control cDC1 (Fig. 2D). Moreover, the frequency of CD80^+^ XCR1^+^ cDC1 was significantly reduced in premorbid B27 rats, before and after GM-CSF treatment (Fig. 2D). In addition, the frequency of RT1B^High^ XCR1^+^ cDC1 was reduced in premorbid HLA-B27^+^ XCR1^+^ cDC1 before GM-CSF treatment (Supplementary Fig. 5). Since decreased CD80 and RT1B expression were already present before stimulation in HLA-B27^+^ XCR1^+^ cDC1, this indicates a direct effect of HLA-B27 expression on XCR1^+^ cDC1 functions linked to T cell activation.

Overall, our results show that GM-CSF exposure enhanced expression of cDC molecules required for their ability to promote T cell proliferation and differentiation and that such GM-CSF response was blunted in HLA-B27^+^ cDCs. Noteworthy, such altered GM-CSF response was particularly pronounced in HLA-B27^+^ XCR1^+^ cDC1 and already present in premorbid B27 rats, highlighting its potential contribution to SpA development in B27 rat.

### GM-CSF signaling in cDCs is principally dependent on JAK2 and JNK

**Fig. 3:**
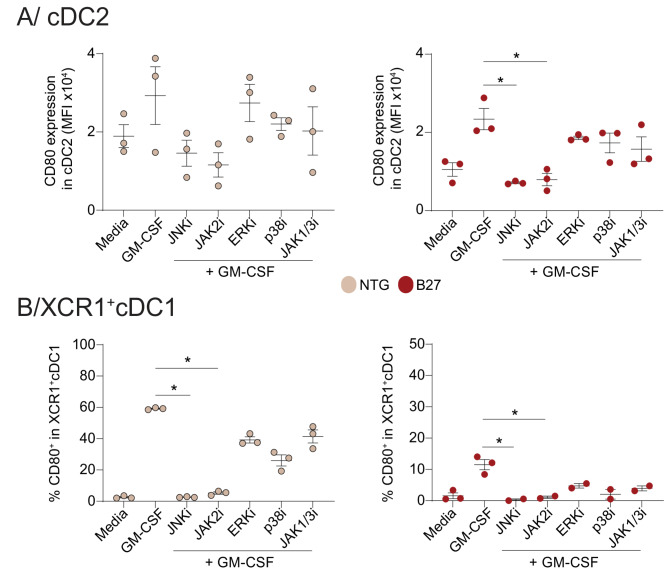
JAK2 and JNK inhibition affects GM-CSF signaling in cDCs. cDC2 and XCR1^+^ cDC1 were isolated from NTG and B27 rats with established SpA. Each subset was stimulated with GM-CSF with or without the following specific inhibitors: Erk (UO126), p38 (SB203580), JNK (SP600125), JAK1/3 (Tofacitinib), and EGFR/JAK2 (AG490). cDCs maturation reflected by CD80 expression levels was evaluated the next day by flow cytometry. The graphs show CD80 expression levels in cDC2 (**A**) and in XCR1^+^ cDC1 (**B**) from NTG (left) and B27 rat (right). The data are normalized to GM-CSF stimulation (Fold change, FC). *n* = 3 rats per group (*: *p* < 0.05)

GM-CSF can regulate through different signaling pathways many cellular functions such as activation and maturation of myeloid cells, including cDCs [13]. To determine if HLA-B27 expression was interfering with one of those pathways, we cultured purified cDC subsets from NTG and B27 rats with specific inhibitors. Inhibition of JNK and JAK2 abrogated CD80 upregulation mediated by GM-CSF in both cDC subsets from NTG rats (Fig. 3, left panels). Similar inhibition was found in HLA-B27^+^ cDCs (Fig. 3, right panels). In contrast, inhibition of ERK, p38 kinase or JAK1/3 affected only partially CD80 upregulation in both cDCs (Fig. 3). Altogether this suggests that the altered GM-CSF response of HLA-B27^+^ cDCs in B27 rats is not specific for one of those pathways.

### GM-CSF-primed cDCs from B27 rat display specific transcriptomic signature

**Fig. 4:**
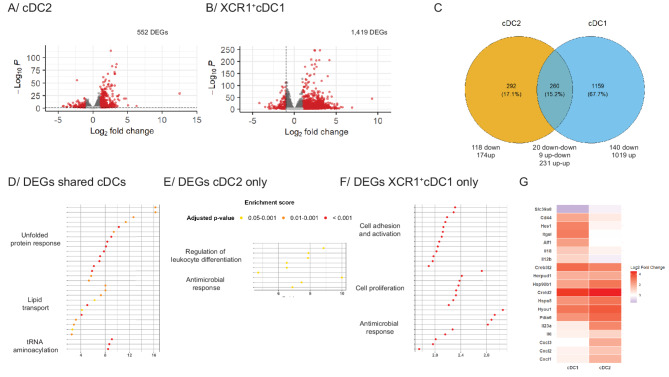
GM-CSF-primed HLA-B27^+^ cDCs transcriptomic signature. cDC2 and XCR1^+^ cDC1 were isolated from NTG and B27 rats with established SpA (*n* = 6 per group) and stimulated with GM-CSF overnight. The next day, RNA was isolated and further sequenced. (**A**-**B**) The volcano plots depict DEGs in cDC2 (**A**) and XCR1^+^ cDC1 (**B**) between NTG and B27 rats. Red dots represent significant DEGs (adjusted p value < 0.05 with a FC > 2) while grey dots represent not differentially expressed genes. (**C**) The Venn diagram represents DEGs shared by cDCs (dark blue) or specific for cDC2 (orange) and XCR1^+^ cDC1 (light blue), respectively. (**D**-**G**) Functional enrichment analysis of DEGs shared by cDCs (**D**), specific for cDC2 (**E**) and specific for XCR1^+^ cDC1 (**F**) were performed. The 30 most deregulated pathways for each list are shown with hierarchical clustering tree and allowed identification of either 2 (cDC2) or 3 (shared cDCs and XCR1^+^ cDC1) clusters. The pathways inside each cluster are sorted according to their respective enrichment score (**G**) The heat-map depicts key DEGs identified between GM-CSF-primed NTG and HLA-B27^+^ cDCs

To better understand which specific pathway(s) might be affected by HLA-B27 expression during rat SpA, we studied the transcriptional program of each cDC subset in response to GM-CSF (Fig. 4). cDC subsets were isolated from adults (> 6 months old) NTG and B27 rats with established SpA and primed overnight as shown in Fig. 2 and RNA was extracted from live cells. We found 552 and 1,419 DEGs between GM-CSF-treated NTG and HLA-B27^+^ cDC2 (Fig. 4A) and XCR1^+^ cDC1 (Fig. 4B), respectively. Among them, only 142 and 169 were downregulated in HLA-B27^+^ cDC2 and XCR1^+^ cDC1, respectively. In contrast, 414 and 1,250 DEGs were upregulated in HLA-B27^+^ cDC2 and XCR1^+^ cDC1, respectively (Fig. 4C and Supplementary Table 1). We further crossed those lists and identified a shared signature of HLA-B27^+^ cDCs as well as cell subset specific transcriptomic signatures in cDC2 and XCR1^+^ cDC1, respectively (Fig. 4C). Importantly, the majority of DEGs concerned HLA-B27^+^ XCR1^+^ cDC1 consistent with predominant functional alterations of those cells (Figs. 1 and 2).

Two hundred and sixty DEGs were shared between both subsets (Fig. 4C). Functional enrichment analysis of DEGs shared by cDCs revealed that endoplasmic reticulum (ER) stress response and unfolded protein response (UPR) were the most significantly enriched pathways in B27 rats, followed by lipid transport and RNA aminoacylation activation (Fig. 4D and Supplementary Fig. 6 and Table 2). *Pdia6*, *Hyou1*, *Hspa5* (coding for BiP), *Hsp90b1*,* Creb*_*3*_*l2* and *Herpud1* were the most representative DEGs involved in the upregulated UPR in B27 rat (Fig. 4G).

In addition to shared DEGs, 292 DEGs were specific for the cDC2 subset (Fig. 4E). The enrichment analysis revealed that regulation of leucocyte differentiation and antimicrobial response were the most overrepresented pathways in B27 rats (Fig. 4E and supplementary Table 3). Thus, several members of the CXC chemokine family (*Cxcl1*,* Cxcl2* and *Cxcl3*), and two proinflammatory cytokine genes, *Il23a* and *Il6*, were strikingly increased in HLA-B27^+^ cDC2 (Fig. 4G), in agreement with their proinflammatory profile (Fig. 1D). These DEGs are consistent with a contribution of HLA-B27^+^ cDC2 to the differentiation bias towards Th17 cells during rat SpA [24].

Finally, the largest set of DEGs was specific of XCR1^+^ cDC1 (1,159 DEGs), affecting several immune pathways linked to cell activation, adhesion and proliferation, and antimicrobial response (Fig. 4F and supplementary Table 4). Cytokine such as *Il12b,Il18* and activation markers such as *Itgal*,* Hes1*, *Aif1*, and *Cd44* were strongly increased in HLA-B27^+^ XCR1^+^ cDC1 (Fig. 4G). In addition, expression of the zinc transporter *Slc39a8*, an NF-κB target inhibiting pro-inflammatory response [25] was decreased in B27 rats (Fig. 4G) suggesting that expression of HLA-B27 leads to dysregulated functions of XCR1^+^ cDC1.

## Discussion

Bridging innate and adaptive immunity, DCs are key to mediate immune tolerance, elicit an immune response and restore tissue homoeostasis. In B27 rats, transfer experiments demonstrated a pathogenic role of CD4^+^ T cells, most likely dependent on HLA-B27^+^ DC dysfunctions. Several DC subsets were discovered in the last 15 years and associated with immune functions from tolerance to strong proinflammatory abilities. During SpA, the contribution of each cDC subset to support proinflammatory T cells differentiation and pathogenicity has not yet been addressed. Furthermore, the production of the growth factor GM-CSF crucial for cDC generation and functions is increased during SpA [16]. The well-established influence of GM-CSF on cDCs led us to evaluate if this cytokine would contribute to the differentiation of proinflammatory T cells during SpA, by impacting cDC functions.

We used the B27 rat model to address this question since functional alterations of cDCs are thought to play a critical role in this model [8, 9, 11, 12, 26]. First, we demonstrated that GM-CSF was required for XCR1^+^ cDC1 functions linked to T cell activation and required for optimal T cell activation by cDC2. Our results are consistent with the recently reported effects of GM-CSF on human cDC1 and cDC2, including increased CD80 and class II-MHC expression and T cell stimulatory capacity [27]. We then compared the function of GM-CSF-primed cDC2 and XCR1^+^ cDC1 between NTG and B27 rat. The proliferation rate of naïve CD4^+^ T cell supported by GM-CSF-primed cDC2 was similarly strong between both rat conditions which is consistent with the comprehensive expression of class II-MHC and CD80 costimulatory molecules on this DC subset, albeit with lower levels in HLA-B27^+^ cDC2. Nevertheless, coculture with the HLA-B27^+^ rat cDC2 resulted in a more proinflammatory phenotype of the divided T cells, as shown by TNF production, which is consistent with previous work [24]. Given that altered functions of cDC2 were only prominent in B27 rats with established disease (Fig. 2), they might be a consequence of this inflammatory state.

In contrast, expression of class II-MHC and CD80/CD86 was not as comprehensive on GM-CSF-primed XCR1^+^ cDC1 from NTG rat which also induced weaker proliferation and TNF production by CD4^+^ T cells than cDC2, in agreement with their tolerogenic function as previously shown [11, 28]. Interestingly however, HLA-B27^+^ XCR1^+^ cDC1 were markedly less responsive to GM-CSF activation as shown by blunted expression class II-MHC and CD80/CD86 and their failure to stimulate T cell proliferation. This was a direct consequence of HLA-B27 expression rather than rat SpA development, as XCR1^+^ cDC1 isolated from healthy nude B27 rats displayed similar altered function. Despite that, XCR1^+^ cDC1 from B27^+^ rats tended to induce heightened TNF production by T cells, similarly to their cDC2 counterpart. Thus, the capacity of cDCs from B27 rats to induce TNF production by T cells appeared inversely related to their expression of activation markers. Noteworthy, we have previously shown defective stimulatory capacity of ex vivo-sorted bulk DCs from B27 rat on naïve CD4^+^ T cells and that the proliferating CD4^+^ T cells were skewed towards a proinflammatory phenotype [5, 8, 29]. We had linked such aberrant behaviors to impaired engagement of costimulatory molecules [5, 8, 9]. This is consistent with the weaker expression levels of those molecules on both subsets of HLA-B27^+^ rat cDCs induced by GM-CSF priming.

To decipher the molecular mechanisms leading to altered response of HLA-B27^+^ cDCs to GM-CSF, we investigated how GM-CSF receptor signals through key downstream pathways involving JAK/STAT and mitogen activated protein kinases [13]. JNK or JAK2 inhibitors completely abolished GM-CSF-induced costimulatory molecule expression on both cDC subsets, pointing out the critical involvement of both pathways in GM-CSF signaling, without differences between B27 and NTG conditions. Thus, HLA-B27 expression appeared to disturb GM-CSF signaling by a molecular mechanism that is not specific for a given pathway. This might not be specific for GM-CSF either, since we observed similar defective response of B27 rat cDCs to toll-like receptors (Amel Ait Ali Said, Chiara Rizzo, Maxime Breban, Simon Glatigny, manuscript in preparation). Among other hypotheses, such widespread alterations could be related to interaction of HLA-B27 with several surface receptors and/or to membrane disorganization induced by expression of this class I-MHC molecule [30–33].

Nevertheless, we uncovered different transcriptional signatures of GM-CSF primed HLA-B27^+^ cDCs. We first identified a signature common to both HLA-B27^+^ cDCs subsets, mostly driven by ER stress and UPR. Outstanding UPR response was evidenced in bone marrow-derived macrophages from B27 rats, obtained after in vitro culture in the presence of macrophage colony-stimulating factor (M-CSF) [34] and our previous transcriptomic and proteomic studies of ex-vivo sorted splenic B27 rat cDCs also exhibited some evidence of UPR [12, 26]. Thus, UPR seems to be a cardinal feature of antigen-presenting cells in B27 rat, maybe due to HLA-B27 misfolding, that is possibly exacerbated upon exposure to M-CSF or GM-CSF [35]. Beyond UPR, GM-CSF-priming induced an HLA-B27^+^-specific signature in each subset of cDC. The genes coding for IL-23 A, that can itself be induced by UPR [36] and IL-6, two cytokines critical for the differentiation of Th17 cells, and several chemokines that attract neutrophils (CXCL1, CXCL2, CXCL6) were increased in HLA-B27^+^ cDC2. This could account for the propensity of those cells to drive a pro-inflammatory Th17 response in B27 rat [24]. The largest number of DEGs between NTG and B27 conditions concerned GM-CSF-primed XCR1^+^ cDC1. Those DEGs were affecting several pathways involved in regulation of cell adhesion, activation, proliferation and antimicrobial response. The upregulation of activation genes linked to proinflammatory profile, such as *Itgal*,* Hes1*,* Cd44* and *Aif*, in HLA-B27^+^ cDC1 is possibly interfering with their tolerogenic functions [37, 38]. In addition, pro-inflammatory cytokine genes, including *Il12b* coding for IL-12p40 shared by IL-12 and IL-23 heterodimers and *Il18* were strongly increased in B27 rat XCR1^+^ cDC1, participating to the inflammatory context. Altogether, this pattern is consistent with the previously reported deficient tolerogenic function of cDC1 in B27 rat [11].

Our finding that cDC1 functions were markedly altered in healthy nude and premorbid B27 rats leads to the hypothesis that SpA development might primarily result from an alteration of the cDC1-mediated tolerance. Hence, intestinal cDC1s are critical to drive tolerance towards gut microbiota, and such essential function could be jeopardized by HLA-B27 expression [39]. This could contribute to intestinal inflammation that is the earliest pathological feature in B27 rat and frequently observed in SpA patients, and consequently to intestinal dysbiosis observed both in B27 rat and patients [40, 41].

Studying the consequence of GM-CSF-priming on B27 rat cDCs allowed us to shed some light on a dual role of GM-CSF in SpA, as in other context [42]. On the one hand, altered cDC2 response to GM-CSF appeared to favor the expansion of proinflammatory CD4^+^ T cells in B27 rat. However, impairment of GM-CSF signaling in B27 rat XCR1^+^ cDC1 might also interfere with Treg induction. This could explain why targeting GM-CSF during SpA was not as efficient as expected [18].

## Electronic supplementary material

Below is the link to the electronic supplementary material.

Supplementary Material 1

Supplementary Material 2

## Data Availability

No datasets were generated or analysed during the current study.
