# Supplementary material for: Granulocyte-macrophage colony-stimulating factor may contribute to spondyloarthritis development in HLA-B27 transgenic rat by affecting conventional dendritic cells function
Source: Arthritis Res Ther. 2025 Jun 13;27:124. doi: 10.1186/s13075-025-03586-9 (PMC12164074; doi:10.1186/s13075-025-03586-9)
Supplement: Supplementary file 2 — Supplementary Material 2 [file 13075_2025_3586_MOESM2_ESM.docx]

**Supplementary Figure 1:** **cDCs and naïve CD4^+^ T cells sorting strategy**. (A) cDC2 (Blue) and XCR1^+^ cDC1 (purple) were sorted from spleens of NTG (top) and B27 rats (bottom). Briefly, single cells were selected, T and B cells contaminants were excluded with anti-TCRαβ and anti-CD45RA Abs respectively. cDC2 (CD103^+^CD4^+^) and cDC1 (CD103^+^CD4^-^) were identified and XCR1 expression on cDC1 was visualized to sort XCR1^+^ cDC1. (B) Naive CD4^+^ T cells were cell sorted from mLN of NTG rats as CD4^+^CD25^-^CD62L^high^.

**Supplementary Figure 2:** **GM-CSF-primed XCR1^+^ cDC1 support Treg differentiation**. cDC2 and XCR1^+^ cDC1 were isolated from NTG rats and cultured overnight with or without GM-CSF. Then, primed cDCs were tested for their ability to support Treg differentiation from NTG naïve CD4^+^ T cells in the presence of anti-TCRαβ mAb. The box and whiskers graph shows the frequency of Tregs (Foxp3^+^) among proliferating T cells after co-culture GM-CSF-primed cDCs. n=9 rats per group * p <0.05.

**Supplementary Figure 3:** **cDC2 from B27 rats support TNF-producing T cells differentiation.** cDC2 were isolated from spleens of adults NTG and B27 rats and primed with GM-CSF overnight. Then, primed cDCs were tested for their ability to support NTG naïve CD4^+^ T cells proliferation and differentiation in the presence of anti-TCRαβ mAb. (A) The representative plot shows T cell proliferation evaluated by CTV dilution and isotype for TNF on live T cells cultured with cDC2 isolated from B27 rat. (B) The representative plots show T cell proliferation evaluated by CTV dilution and TNF production on live T cells cultured with cDC2 isolated from NTG and B27 rat.

**Supplementary Figure 4: Similar CD80 expression levels on cDCs subsets from NTG, B27 and B7 rats *ex vivo*.** Spleens from adults NTG, B7 and B27 rats were collected, and cDCs isolated. (A) CD80 expression levels were determined by flow cytometry on XCR1^+^ cDC1 and cDC2. The graphs show *ex vivo* CD80 expression levels on cDC2 (left) and cDC1 (right) from NTG, B27 and B7 rats. (B) The representative plots show FMO (filled grey) and CD80 expression (blue line) on XCR1^+^ cDC1 from NTG rats after overnight GM-CSF priming.

**Supplementary Figure 5:** **Altered expression of CD86 and RT1-B in B27 rat cDCs in response to GM-CSF.** cDC2 (A,B) and XCR1^+^ cDC1 (C,D) were isolated from adult NTG, B27 (with established SpA) and B7 rats or 3-weeks-old NTG and B27 premorbid rats and primed in absence (-) or presence of GM-CSF overnight (extended figure 2). CD86 (A,C) and RT1B (B,D) expression levels were evaluated the next day by flow cytometry. The expression of RT1B and CD86 were determined according to FMO staining. The graphs show the mean fluorescence intensity (MFI) of CD86 and RT1B expression in cDC2 and the frequency of CD80^+^ cells and RT1B^High^ among XCR1^+^ cDC1 from NTG (beige), B27 (red) and B7 (magenta) rats. Results are shown as box and whiskers graph. n= 5 rats per group; *: p<0.05

**Supplementary Figure 6:** **Altered cDC transcriptomic signature in HLA-B27 rats.** cDC2 and XCR1^+^ cDC1 were isolated from NTG and B27 rats with established SpA (n=6 per group) and stimulated with GM-CSF overnight. The next day, RNA was isolated and further sequenced. (A-C) Functional enrichment analysis of DEGs shared by cDCs (A), specific for cDC2 (B) and specific for XCR1^+^ cDC1 (C) were performed. The 30 most deregulated pathways for each list are shown with hierarchical clustering tree and allowed identification of either 2 (cDC2) or 3 (shared cDCs and XCR1^+^ cDC1) clusters. The pathways inside each cluster are sorted according to their respective enrichment score.
